# *Thra* knockout protects male mice from hyperthyroidism-driven cortical bone loss by mitigating bone resorption

**DOI:** 10.1093/jbmrpl/ziag033

**Published:** 2026-03-06

**Authors:** Franziska Brinkmann, Eleonore Kreß, Christiane Schirm, Lorenz C Hofbauer, Martina Rauner, Elena Tsourdi

**Affiliations:** Department of Medicine III and Center for Healthy Aging, Medical Faculty and University Hospital Carl Gustav Carus, Dresden University of Technology, 01307 Dresden, Germany; Department of Medicine III and Center for Healthy Aging, Medical Faculty and University Hospital Carl Gustav Carus, Dresden University of Technology, 01307 Dresden, Germany; Department of Medicine III and Center for Healthy Aging, Medical Faculty and University Hospital Carl Gustav Carus, Dresden University of Technology, 01307 Dresden, Germany; Department of Medicine III and Center for Healthy Aging, Medical Faculty and University Hospital Carl Gustav Carus, Dresden University of Technology, 01307 Dresden, Germany; Department of Medicine III and Center for Healthy Aging, Medical Faculty and University Hospital Carl Gustav Carus, Dresden University of Technology, 01307 Dresden, Germany; Department of Medicine III and Center for Healthy Aging, Medical Faculty and University Hospital Carl Gustav Carus, Dresden University of Technology, 01307 Dresden, Germany

**Keywords:** hyperthyroidism, thyroid hormone receptor alpha, *Thra*, osteoporosis, cortical bone, bone, resorption, osteocyte, osteoclast

## Abstract

Secondary osteoporosis is a major global health problem and can be caused by endocrine diseases, including hyperthyroidism. Of note, hyperthyroid mice exhibit both trabecular and cortical bone loss, accompanied by reduced bone strength, as a consequence of a high bone turnover in which bone resorption exceeds bone formation. Recently, we discovered that osteocytes in bones obtained from hyperthyroid mice develop osteoclast-like features. However, the role of thyroid hormone receptor α (TRα) in hyperthyroidism-driven bone resorption remains ill-defined. Here, we rendered male *Thra*^0/0^ mice (which lack all TRα isoforms) hyperthyroid, and comprehensively characterized their bone phenotype. *Thra*^0/0^ mice with L-thyroxine (T4) treatment displayed trabecular, but not cortical bone loss, and maintained bone strength, while T4-treated WT littermates showed overall reduced bone mass and strength. Serum levels of bone resorption marker tartrate-resistant acid phosphatase (TRAP) and numbers of TRAP-positive osteoclasts were elevated in hyperthyroid WT, but not *Thra*^0/0^ mice. We also detected increased numbers of TRAP-positive osteocytes in long bones of T4-treated WT mice. In line, the expression of osteoclast marker genes was upregulated with T4 in bone tissue from WT mice only. While thyroid hormones (THs) have been reported not to directly affect osteoclast activity, our in vitro studies using late osteoblasts/osteocytes derived from *Thra*^0/0^ and WT mice revealed that the TH-induced expression of osteoclast markers in these cells was *Thra*-dependent. Further, the *Rankl/Opg* ratio linking osteocytes to enhanced osteoclastogenesis was upregulated in hyperthyroid WT, but not *Thra*^0/0^ cells. Taken together, we demonstrate that *Thra* KO can preserve cortical bone mass and bone strength by obstructing an increase of bone-resorbing osteoclasts and osteocytes with osteoclast-like features in hyperthyroid male mice.

## Introduction

Osteoporosis is a major global public health concern, with osteoporotic fractures contributing substantially to both morbidity and mortality. Although primary postmenopausal osteoporosis is most common, secondary osteoporosis is observed in up to 30% of postmenopausal women, over 50% of premenopausal women, and between 50% and 80% of men.^1^^,^^2^ Overt hyperthyroidism is one of the established causes of secondary osteoporosis.^3^^,^^4^ In the adult skeleton, systemic excess of the thyroid hormones (TH) L-thyroxine (T_4_) and 3,5,3′-triiodo-L-thyronine (T_3_) leads to an accelerated bone turnover with enhanced osteoblast and osteoclast activity. Given the prevalent osteoclast-mediated bone resorption, a net bone loss is observed that adds to bone fragility and increased fracture risk.^5–8^

Distinct mouse models with exogenously added THs confirmed a pronounced decrease in trabecular and cortical bone mass with impaired bone strength.^9–12^ Increased osteoblast numbers and high bone formation rate co-occurring with high osteoclast numbers in bone tissue as well as elevated serum concentrations of osteoclast markers tartrate-resistant acid phosphatase (TRAP) corroborate the high bone turnover state that causes osteoporosis in hyperthyroid mice.^9–12^

To exert their physiological actions in bone, TH can bind to 3 functional TH receptors (TR), namely TRα1, TRα2, and TRβ1 that are encoded by the genes *Thra* and *Thrb.*^13^^,^^14^ Additional isoforms are expressed in bone, such as TRΔα1 and TRΔα2, however, they fail to bind TH and may act as antagonists.^3^^,^^13^ In the periphery, T_4_ is mostly converted by deiodinase 1 (DIO1) and 2 (DIO2) into T_3_ given that the TR-binding affinity of T_3_ is 15-fold higher than that of T_4_.^3^

Predominantly TRα1 is expressed in bone tissue and several studies using distinct *Thra* mutant mice have highlighted its important role in skeletal development and bone maintenance.^3^^,^^15–20^

In this study, we use *Thra*^0/0^ mice that express no TRα isoforms, but show normal *Thrb* expression. *Thra*^0/0^ mice are euthyroid with normal circulating T_4_, T_3_, and thyroid-stimulating hormone levels.^3^^,^^21^ While *Thra*^0/0^ juveniles exhibit a transient growth delay, retarded endochondral ossification and abated mineral deposition, adult *Thra*^0/0^ mice display a trabecular bone remodeling defect leading to osteosclerotic bones as a consequence of absent TRα1-mediated TH action in bone (“local hypothyroidism”).^3^^,^^21^

At the cellular level, TH directly stimulate the differentiation and function of osteoblasts.^22–28^ In contrast, osteoclasts and their resorptive activity appear to be influenced primarily through indirect mechanisms mediated by TH-responsive cells of the osteoblastic lineage in their vicinity.^3^^,^^9^^,^^29–33^ In our recent study, we demonstrated that a *Thra* KO enhanced the differentiation and activity of early, mature, and late osteoblasts under cell culture conditions.^18^ Nevertheless, *Thra*-deficient osteoblasts remain TH responsive in vitro leading to an even more pronounced osteoblast activity when treated with TH as compared to TH-treated WT cells.^18^

While the actions of TH and *Thra* in osteoblasts have been well-described, little is known about their functions in osteocytes, the bone-embedded mechanosensory cells that direct bone remodeling. Various cytokines secreted by osteocytes regulate the coupling between osteoblasts and osteoclasts. Notably, the RANKL, a key stimulator of osteoclastogenesis, and osteoprotegerin (OPG), a strong decoy receptor for RANKL that inhibits osteoclast activity, serve as critical mediators linking osteocyte function and bone resorption.^34^^,^^35^ Our recent study on hyperthyroid mice revealed that osteocytes can also acquire osteoclast-like characteristics, such as TRAP activity, and actively remodel their surrounding lacunae and thus contribute to bone remodeling,^10^ a phenomenon referred to as osteocytic osteolysis.^36–38^ Under high bone turnover conditions, such as lactation, hyperparathyroidism, and hyperthyroidism, osteocytic osteolysis can directly contribute to overall bone loss.^10^^,^^39^^,^^40^ Still, it is not known whether these TH actions are directly mediated by TR in osteocytes.

Here, we rendered male *Thra*^0/0^ mice hyperthyroid to unravel the role of *Thra* in hyperthyroidism-driven bone resorption.

## Materials and methods

### Animal models

For our in vivo studies, we used adult male *Thra*^0/0^ mice on C57BL/6 background and respective age and sex-matched WT (*Thra*^wt/wt^, internal abbreviation WT) littermates as controls that were a kind gift from Prof. Lars Möller (University Hospital Essen). Mice with a homozygous mutation of *Thra*^0/0^ do not express any *Thra* isoforms as described previously.^21^^,^^41^ Mice were maintained in groups up to 4 animals in a light–dark cycle of 12/12 h at room temperature in filter-top cages with cardboard houses for enrichment purposes and had ad libitum access to their respective drinking water and standard chow diet. Genotyping was performed by PCR analysis. To induce hyperthyroidism, 12-wk-old male *Thra*^0/0^ and WT mice were treated over 4 wk with 1.2 μg/mL L-thyroxine (T4, Sigma-Aldrich) in their drinking water. Control littermates received regular tap water (CO). Group sizes were as follows: micro-CT: WT mice: CO: *N* = 10; T4: *N* = 10; *Thra*^0/0^ mice: CO: *N* = 9; T4: *N* = 10; compression test: WT mice: CO: *N* = 7; T4: *N* = 10; *Thra*^0/0^ mice: CO: *N* = 7; T4: *N* = 10; 3-point bending test: WT mice: CO: *N* = 10; T4: *N* = 9; *Thra*^0/0^ mice: CO: *N* = 9; T4: *N* = 9; ELISA: WT mice: CO: *N* = 9; T4: *N* = 8; *Thra*^0/0^ mice: CO: *N* = 9; T4: *N* = 9. Real-time qPCR analysis of bone tissue RNA: WT mice: CO: *N* = 10; T4: *N* = 10; *Thra*^0/0^ mice: CO: *N* = 7; T4: *N* = 7. At an age of 16 wk, all mice were euthanized using CO_2_. Blood was collected via heart puncture and serum was obtained by centrifugation. The LS, femurs, and tibias were collected postmortem, fixed in 4% PBS-buffered paraformaldehyde for 48 h, and stored in 50% ethanol. For RNA isolation of bone tissue, humeri were flushed with PBS, immediately frozen with liquid nitrogen and stored at −80 °C until further analysis. All subsequent analyses (ELISA, micro-CT, bone biomechanics, histology/dynamic histomorphometry, and RNA analysis of bone tissue) were performed in a blinded manner.

Animal procedures were approved by the Institutional Animal Care Committee of the Technische Universität Dresden and the Landesdirektion Sachsen (TVV 09/2021) and were performed according to the ARRIVE guidelines. We have complied with all relevant ethical regulations for animal use.

### Serum analysis

Serum concentrations of bone resorption marker TRAP and bone formation marker procollagen type 1 amino-terminal propeptide (P1NP) were assessed using ELISAs according to the manufacturers’ protocols (IDS GmbH).

### Analysis of bone mass and microarchitecture

Femurs and fourth lumbar vertebrae (L4) were analyzed using micro-CT (vivaCT 40, Scanco Medical) with an X-ray energy of 70 kVp and isotropic voxel size of 10.5 μm (114 mA, 200 ms integration time). Trabecular (Tb.) and cortical (Ct.) bone parameters, including bone volume/total volume (BV/TV), trabecular number (Tb.N), trabecular separation (Tb.Sp), and thickness (Tb.Th), were evaluated based on calculations including 100 scan slices following standard protocols from Scanco Medical. Trabecular bone analysis of L4 was performed at the center contouring 50 slices above and 50 slices below the middle of the vertebral body. Femur trabecular bone parameters were conducted in the metaphyseal region starting 20 slices below the growth plate, while cortical bone was assessed within the diaphyseal region midway between femoral head and distal condyles.

### Static and dynamic histomorphometry

Following fixation in 4% PBS-buffered paraformaldehyde for 48 h, L4 vertebrae and femurs were decalcified in Osteosoft (Merck) for 7 d and dehydrated using ascending ethanol series. Then, decalcified bones were embedded in paraffin and cut into 2-μm-thick sections. Tartrate-resistant acid phosphatase staining was performed to quantify osteoclasts in an area of 0.90 and 0.48 mm^2^ in the center of vertebrae and femoral midshaft, respectively, using the Microscope Axio Imager M1 (Carl Zeiss Jena) and Osteomeasure software (OsteoMetrics). In addition, trabecular TRAP-positive osteocytes (Tb.TRAP+ Ot) were identified by their staining and localization within the bone tissue and quantified in an area of 0.90 and 0.48 mm^2^ in the center of vertebrae and femoral midshaft, respectively. To evaluate the amount of cortical TRAP+ osteocytes (Ct.TRAP+ Ot), 1-200 osteocytes were analyzed within the in cortical bone of femurs as described previously.^10^ TRAP+ osteocytes were normalized to the total number of evaluated osteocytes (Ot.N). Representative photos were taken using CellSens Entry Software Version 1.5 (OLYMPUS Cooperation).

Dynamic bone histomorphometry was conducted as described previously.^9^^,^^12^ Five and two days before sacrifice, mice received intraperitoneal injections with the fluorochrome calcein (20 mg/kg BW; Sigma-Aldrich) that incorporates into newly formed bone. The third lumbar vertebrae (L3) and tibiae were fixated in 4% PBS-buffered paraformaldehyde for 48 h and dehydrated via ascending ethanol series. Subsequently, bones were embedded in methyl methacrylate (Technovit 9100, Heraeus Kulzer) and cut into 7 μm sagittal sections for calcein label quantification of the trabecular bone. The mineralized surface/bone surface (MS/BS), the mineral apposition rate (MAR), and the bone formation rate/bone surface (BFR/BS) were quantified in an area of 1.44 and 0.72 mm^2^ in the center of vertebrae and tibial midshaft according to established protocols^9^^,^^10^^,^^42–44^ using fluorescence microscopy (Microscope Axio Imager M1) and the Osteomeasure software. Representative photos were taken using Axio Vision 3.1 Software (Carl Zeiss Jena). Terminology and quantification procedures were conducted according to guidelines of the Nomenclature Committee of the ASBMR.^45^

### Bone biomechanics

The fifth lumbar vertebrae (L5) were tested using a compression test (Zwick Roell) while femurs were used for 3-point bend testing to assess cortical bone strength. Beforehand, femurs and L5 vertebrae were rehydrated in PBS overnight. Vertebrae were positioned onto the center of the lower plate and pressure was applied via the upper plate. Femurs were placed onto 2 supports with an intermediate distance of 6 mm and a mechanical force was applied vertically onto the middle of the femoral midshaft. After reaching a preload of 1 N, the measurement started and continued with a load rate of 0.05 mm/s until failure. The maximal load (F_max_) was evaluated as an indicator of bone strength using testXpert II—V3.7 software (Zwick Roell).

### Cell culture of primary murine late osteoblasts/osteocytes

Hind legs of *Thra*^0/0^ and WT mice were collected and mesenchymal stromal cells (MSC) were obtained by flushing the bone marrow. To acquire primary murine osteoblasts, MSC were first cultured in growth medium (DMEM, 10% FCS, 1% Penicillin/Streptavidin (P/S)) until 80% confluence in a humidified atmosphere of 95% air and 5% CO_2_ at 37 °C. Subsequently, MSC were differentiated into late osteoblasts/osteocytes by adding an osteogenic cocktail (100 μM ascorbate phosphate, 5 mM β-glycerol phosphate, both from Sigma-Aldrich) to the growth medium over 25 d. For RNA analysis, osteoblasts were then starved overnight in DMEM with 1% FCS and 1%P/S and treated the next day with 100 nM T_3_ (Sigma-Aldrich) over 48 h.

To determine the mineralization capacity, MSC were differentiated and simultaneously treated with 100 nM T_3_ over 28 d, then fixed in 10% paraformaldehyde in PBS for 15 min and stained with 1% Alizarin Red S solution (pH 5.5, Sigma-Aldrich) for 30 min at room temperature. To remove excess stain, repeated washing steps with distilled water were performed. 100 mM cetylpyridinium chloride solution (Sigma-Aldrich) was used to dissolve incorporated calcium that was then quantified by photometric measurement at a wavelength of 540 nm using FluoStar Omega (BMG Labtech). Each in vitro experiment was performed at least twice with a number of >4 animals per group. All subsequent analyses were conducted in a blinded fashion.

### RNA isolation, RT-PCR, and quantitative real-time PCR

Total RNA from primary cells and bone tissue was extracted using TRIzol reagent (Invitrogen) following the manufacturer’s protocol and quantified using a Nanodrop spectrophotometer (Peqlab).

Five hundred nanograms of RNA were reverse transcribed using M-MLV Reverse Transcriptase (Promega) followed by GoTaq qPCR Master Mix-based quantitative real-time PCR (Promega) according to established protocols (ABI7500 Fast, Applied Biosystems). Primer sequences for mice are listed in Table S1. PCR conditions were: 50 °C for 5 min and 95 °C for 10 min followed by 40 cycles with 95 °C for 15 s and 60 °C for 1 min. Melting curves were evaluated using the following scheme: 95 °C for 15 s, 60 °C for 1 min, and 95 °C for 30 s. Results were calculated based on the ∆∆CT method and are represented as x-fold increase normalized to β-actin mRNA levels.

### Statistical analysis

Data are presented as mean ± SD. Statistical analysis comparing 4 groups are based on a 2-way ANOVA followed by Bonferroni’s multiple comparison post-hoc test using GraphPad Prism 10 (GraphPad). Values of *p* < .05 were considered statistically significant. Significant outliers were excluded based on Grubbs’ test provided in GraphPad by Dotmatics (https://www.graphpad.com/quickcalcs/grubbs1/).

## Results

### *Thra* KO rescues cortical bone mass and bone strength in hyperthyroid male mice

**Figure 1:**
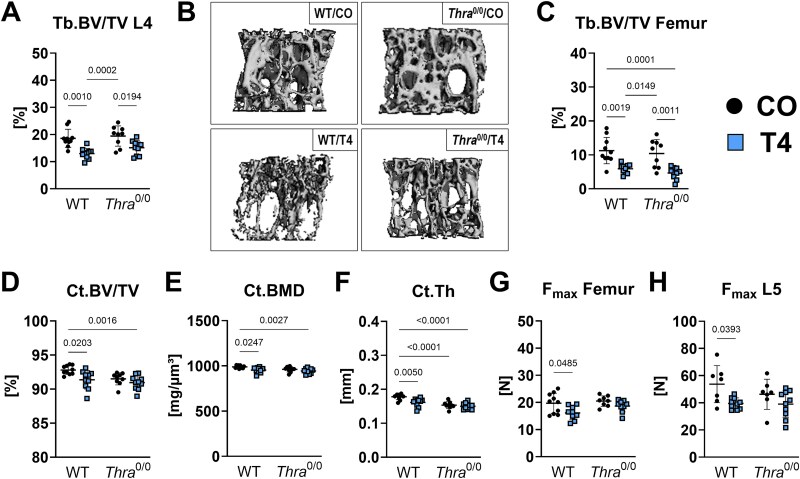
*Thra* deletion does not prevent hyperthyroidism-induced trabecular bone loss, but preserves cortical bone volume and strength. Twelve-week-old male *Thra* KO mice (*Thra*^0/0^) and respective WT littermates remained euthyroid (CO) or were rendered hyperthyroid (T4) by adding 1.2 μg/mL L-thyroxine into their drinking water over 4 wk. Using micro-CT analysis, (A) trabecular bone volume per total volume (Tb.BV/TV) was determined at the fourth lumbar vertebra (L4). (B) Representative pictures of the L4 scans. Additionally, (C) Tb.BV/TV was assessed at the femur. At the femoral midshaft, (D) cortical bone volume over total volume (Ct.BV/TV), (E) cortical BMD (Ct.BMD), and (F) cortical thickness (Ct.Th) were quantified. The maximal force (F_max_) as an indicator of bone strength (G) at the L5 vertebra and (H) femur was tested by compression test and 3-point bending test, respectively. Each dot indicates an individual mouse. Micro-CT: WT/CO: *N* = 10; WT/T4: *N* = 10; *Thra*^0/0^/CO: *N* = 9; *Thra*^0/0^/T4: *N* = 10; compression test: WT mice: CO: *N* = 7; T4: *N* = 10; *Thra*^0/0^ mice: CO: *N* = 7; T4: *N* = 10; 3-point bending test: WT mice: CO: *N* = 10; T4: *N* = 9; *Thra*^0/0^ mice: CO: *N* = 9; T4: *N* = 9. The horizontal lines represent the mean ± SD. Statistical analysis was performed by 2-way ANOVA and *p*-values are shown within the graph.

To investigate the effects of TH excess on bone mass, microarchitecture, and strength in dependence of *Thra* expression, we subjected *Thra*^0/0^ mice and age/sex-matched WT controls to T4 treatment over 4 wk. Micro-CT analysis revealed significant trabecular bone volume loss at the spine and femur of both, WT and *Thra*^0/0^ mice with hyperthyroidism (Tb.BV/TV L4: WT: −30.18%; *Thra*^0/0^: −22.16%, Tb.BV/TV femur: WT: −47.50%; *Thra*^0/0^: −55.41%, Figure 1A-C). Further, we found a comparable BMD loss in the femur and L4 of hyperthyroid WT and *Thra*^0/0^ mice (Table 1). Trabecular numbers were significantly decreased in femurs of T4-treated WT and *Thra*^0/0^ mice (Tb.N femur: WT: −25.05%; *Thra*^0/0^: −17.90%, Table 1). In line, trabecular separation in femurs was increased with hyperthyroidism in both groups (Tb.Sp Femur: WT: +135.56%; *Thra*^0/0^: +124.64%, *p* = .0671, Table 1). These data point to a TH excess-driven osteoporotic phenotype within the trabecular bone compartment regardless of *Thra* expression (Table 1). In contrast, hyperthyroidism led to a significant decrease of cortical bone volume (WT/T4 −1.54% vs WT/CO), BMD (WT/T4 −3.70% vs WT/CO), and thickness (WT/T4 −9.41% vs WT/CO) in WT, but not *Thra*^0/0^ mice implying a site-specific role of *Thra* in bone (Figure 1D-F). Evaluating our data from the 3-point bending test of the femurs and compression test of the L5 vertebrae, we found that maximum strength at both sites was preserved in *Thra*^0/0^ mice despite T4 treatment (Figure 1G and H). Thus, we identify TRα as an important mediator of TH actions in cortical bone.

**Table 1:** Body weight and selected bone parameters of male *Thra*^0/0^ mice and respective control animals.

**Parameter**	**WT/CO**	**WT/T4**	***p*-value** **WT/CO vs WT/T4**	***Thra*** ^**0/0**^ **/CO**	***Thra*** ^**0/0**^ **/T4**	***p*-value** ***Thra*** ^**0/0**^ **/CO** **vs *Thra*** ^**0/0**^ **/T4**	***p*-value** **WT/CO** **vs *Thra*** ^**0/0**^ **/CO**
**Body weight [g]**	29.63 ± 1.77	30.24 ± 2.41	>.999 9	25.2 ± 0.64	25.82 ± 1.31	>.9999	**<.0001**
**BMD L4 [mg/cm** ^**3**^ **]**	212.49 ± 25.21	170.77 ± 12.82	**.0004**	230.34 ± 25.69	205.1 ± 16.43	.0700	.4092
**Tb.N L4 [1/mm]**	4.48 ± 0.46	4.05 ± 0.31	.1483	4.86 ± 0.41	4.86 ± 0.42	>.9999	.2529
**Tb.Th L4 [mm]**	0.050 ± 0.006	0.046 ± 0.004	.1659	0.046 ± 0.005	0.041 ± 0.003	.0807	.1893
**Tb.Sp L4 [mm]**	0.22 ± 0.02	0.25 ± 0.02	**.0249**	0.20 ± 0.01	0.21 ± 0.02	>.9999	.1961
**BMD femur [mg/cm** ^**3**^ **]**	158.47 ± 36.60	101.30 ± 14.56	**.0001**	173.81 ± 29.10	116.90 ± 19.21	**.0002**	>.999
**Tb.N femur [1/mm]**	4.39 ± 0.56	3.29 ± 0.44	**.0005**	5.57 ± 0.58	4.57 ± 0.61	**.0022**	**.0003**
**Tb.Th femur [mm]**	0.043 ± 0.003	0.042 ± 0.004	>.9999	0.037 ± 0.003	0.031 ± 0.001	**.0054**	**.0002**
**Tb.Sp femur [mm]**	0.23 ± 0.03	0.31 ± 0.05	**<.0001**	0.18 ± 0.02	0.22 ± 0.04	.0671	**.0292**
**Oc.N/B.Pm L4 [1/mm]**	12.53 ± 3.67	17.80 ± 5.87	.085	10.87 ± 3.20	16.49 ± 3.64	.1396	>.999
**Oc.N./B.Pm femur [1/mm]**	8.38 ± 3.42	17.64 ± 6.84	**.0009**	7.93 ± 2.70	14.50 ± 4.23	.1566	>.999
**MS/BS L3 [%]**	27.22 ± 4.64	29.74 ± 6.58	>.9999	21.71 ± 4.83	27.75 ± 2.46	.2499	.2179
**MAR L3 [μm/d]**	1.43 ± 0.16	1.51 ± 0.27	>.9999	1.31 ± 0.19	1.29 ± 0.24	>.9999	>.9999
**BFR tibia [μm** ^**3**^ **/μm** ^**2**^ **/d]**	0.29 ± 0.13	0.38 ± 0.15	.8718	0.34 ± 0.11	0.50 ± 0.13	.1907	>.9999
**MS/BS tibia [%]**	19.57 ± 5.58	21.68 ± 4.33	>.9999	23.66 ± 6.02	30.48 ± 4.62	.1137	.7507
**MAR tibia [μm/d]**	1.36 ± 0.29	1.79 ± 0.54	.0814	1.45 ± 0.21	1.63 ± 0.24	>.9999	>.9999
**Tb.Ot.N/B.Ar L4 [1/mm** ^**2**^ **]**	551.71 ± 64.13	530.87 ± 154.60	>.9999	682.11 ± 72.21	623.65 ± 150.67	>.9999	.1971
**TRAP+Ot L4 [%]**	1.47 ± 0.98	2.11 ± 1.51	>.9999	1.35 ± 1.14	0.83 ± 0.71	>.9999	>.9999
**Tb.Ot.N/B.Ar femur [1/mm** ^**2**^ **]**	595.58 ± 137.58	597.88 ± 181.23	>.9999	778.94 ± 127.23	698.57 ± 189.23	>.9999	.1675
**Ct.Ot.N/B.Ar femur [1/mm** ^**2**^ **]**	545.60 ± 99.01	518.19 ± 103.77	>.9999	633.26 ± 37.74	625.97 ± 86.62	>.9999	.3317

Twelve-week-old male *Thra* KO (*Thra*^0/0^) and respective WT (*Thra*^wt/wt^, internal abbreviation WT) mice remained euthyroid (CO) or were rendered hyperthyroid (T4) by adding 1.2 μg/mL L-thyroxine into their drinking water over 4 wk. Micro-CT analysis of the fourth lumbar vertebra (L4) and distal femur were used to determine BMD, trabecular number (Tb.N), trabecular separation (Tb.Sp), trabecular thickness (Tb.Th), as well as cortical BMD (Ct.BMD) and cortical thickness (Ct.Th). Using static and dynamic histomorphometry analyses of the L4/L3 vertebra, femoral midshaft, and distal tibia, osteoclast number per bone perimeter (Oc.N./B.Pm), total number of osteocytes over bone area (Ot.N/B.Ar), percentage of TRAP+ osteocytes at the spine (TRAP+ Ot. L4) mineralized bone surface over bone surface (MS/BS), mineral apposition rate (MAR), and bone formation rate (BFR) were quantified. Mean ± SD are indicated for each group. Micro-CT: WT/CO: *N* = 10; WT/T4: *N* = 10; *Thra*^0/0^/CO: *N* = 9; *Thra*^0/0^/T4: *N* = 10; histology/dynamic histomorphometry: WT/CO: *N* = 10; WT/T4: *N* = 10; *Thra*^0/0^/CO: *N* = 7; *Thra*^0/0^/T4: *N* = 7. Statistical analysis was performed by 2-way ANOVA.

The bold values in Table 1 represent the *p*-values indicating statistical significance

### Global *Thra* deficiency prevents an increase in TRAP-expressing osteoclasts and osteocytes with hyperthyroidism

**Figure 2:**
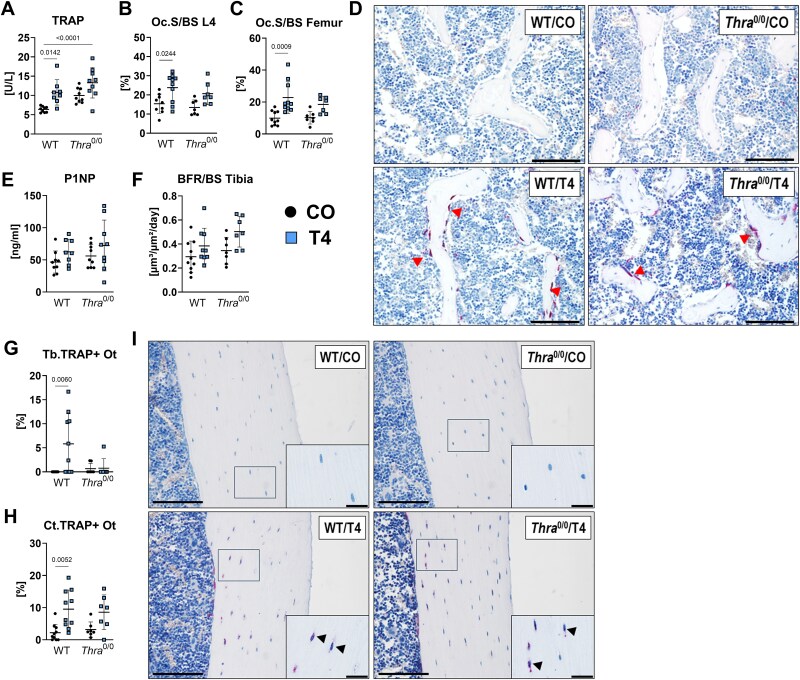
*Thra* loss hampers the increase of TRAP-expressing osteoclasts and osteocytes with TH excess. Twelve-week-old male *Thra* KO mice (*Thra*^0/0^) and respective WT littermates remained euthyroid (CO) or were rendered hyperthyroid (T4) by adding 1.2 μg/mL L-thyroxine into their drinking water over 4 wk. To assess bone resorption, (A) serum concentrations of bone resorption marker TRAP were quantified by ELISA and osteoclast surface over bone surface (Oc.S/BS) was assessed at (B) the spine and (C) distal femur. (D) Representative pictures of TRAP-stained trabecular bone in the distal femur. Furthermore, (E) bone formation marker P1NP was tested in the serum and (F) the bone formation rate (BFR) was evaluated by dynamic histomorphometry in tibias. Using histological slides of the distal femur and femoral midshaft, TRAP-positive osteocytes (TRAP+ Ot) were counted at the (G) trabecular (Tb.) and (H) cortical bone (Ct.) compartment and normalized to the respective total number of osteocytes. (I) Representative pictures of TRAP-stained cortices. Scale bar = 100 μm. Scale bar in magnified images of osteocytes (Fi. 2 I) = 20 μm. The red arrows point to TRAP+ osteoclasts, while black arrows indicate TRAP+ osteocytes. Each dot indicates an individual mouse. Histology: WT/CO: *N* = 10; WT/T4: *N* = 10; *Thra*^0/0^/CO: *N* = 7; *Thra*^0/0^/T4: *N* = 7; ELISA: WT/CO: *N* = 9; WT/T4: *N* = 8; *Thra*^0/0^/CO: *N* = 9; *Thra*^0/0^/T4: *N* = 9. The horizontal lines represent the mean ± SD. Statistical analysis was performed by 2-way ANOVA and *p*-values are shown within the graph.

Given the prevalent bone resorption leading to a net bone loss in hyperthyroid mice, we first checked osteoclast parameters in serum and bone tissue in T4- vs untreated groups. Concentration of serum TRAP was significantly elevated in WT mice due to T4 treatment (WT: +169.41%), however, not in *Thra*^0/0^ mice (*Thra*^0/0^: +133.15%, *p* = .095, Figure 2A). Osteoclast surface and number were also increased at the spine and femur with hyperthyroidism in WT mice only (Oc.S/BS L4: WT: +154.19%, Oc.S/BS femur: WT: +229.99%, Figure 2B-D, Table 1) implying that *Thra* loss in osteoclasts themselves and/or osteoclast-influencing cells in the nearby surrounding might mitigate hyperthyroidism-driven bone resorption. With regards to bone formation, neither serum levels of P1NP nor dynamic histomorphometry parameters were changed within WT and *Thra*^0/0^ cohorts with T4 (Figure 2E and F, Table 1). While the total number of osteocytes did not significantly differ between the groups (Table 1), we counted more TRAP-expressing (TRAP+) osteocytes in the trabecular and cortical compartment of hyperthyroid WT femurs (Tb.TRAP+ Ot: WT: +583.0%, Ct.TRAP+ Ot: WT: +429.1%), but not long bones derived from hyperthyroid *Thra*^0/0^ mice (Tb.TRAP+ Ot: *Thra*^0/0^: +114.46%, *p* > .999, Ct.TRAP+ Ot: *Thra*^0/0^: +268.82%, *p* = .18, Figure 2G-I). Given high standard deviations, we did not detect significant differences in the number of vertebral TRAP+ osteocytes comparing the four groups (Table 1). Overall, these data point to *Thra* as a regulator of TRAP activity in osteoclasts and osteocytes.

### Osteoclast marker expression is upregulated in bone tissue from hyperthyroid WT but not *Thra*-deficient mice

**Figure 3:**
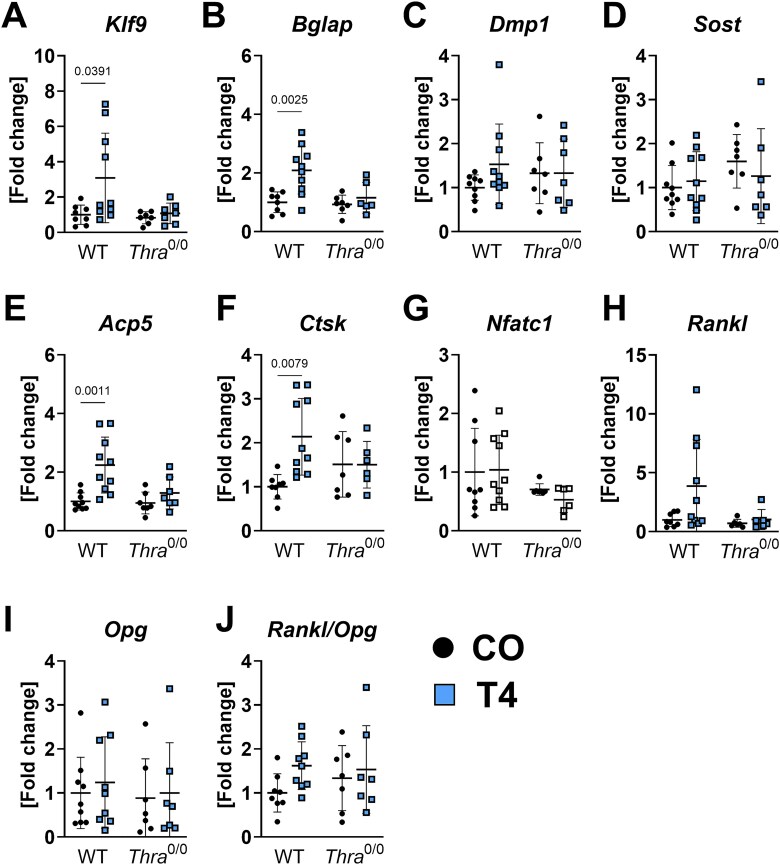
Expression of osteoclast markers is upregulated by T4 only in WT mice. Twelve-week-old male *Thra* KO mice (*Thra*^0/0^) and respective WT littermates remained euthyroid (CO) or were rendered hyperthyroid (T4) by adding 1.2 μg/mL L-thyroxine into their drinking water over 4 wk. Total bone tissue RNA was isolated and tested regarding the expression of (A) krueppel like factor 9 (*Klf9*), (B) osteocalcin/bone gamma-carboxyglutamate protein (*Bglap*), (C) dentin matrix acidic phosphoprotein 1 (*Dmp1*), (D) sclerostin (*Sost*), (E) TRAP-encoding gene acid phosphatase 5, tartrate resistant (*Acp5*), (F) cathepsin K (*Ctsk*), (G) nuclear factor of activated T-cells cytoplasmic 1 (*Nfatc1*), (H) receptor activator of NF-κB ligand (*Rankl*), and (I) osteoprotegerin (*Opg*) using real-time quantitative PCR analysis. Further, (J) the *Rankl/Opg* ratio was calculated. Each dot indicates an individual mouse. WT/CO: *N* = 10; WT/T4: *N* = 10; *Thra*^0/0^/CO: *N* = 7; *Thra*^0/0^/T4: *N* = 7. The horizontal lines represent the mean ± SD. Statistical analysis was performed by 2-way ANOVA and *p*-values are shown within the graph.

To screen for gene expression patterns in reaction to T4 treatment, we used total RNA isolates from bones of all 4 groups. Thyroid hormone target gene *Klf9* was increased by 3.09-fold in bone tissue with hyperthyroidism in WT, but not *Thra*^0/0^ mice indicating a possibly impaired TH response in bone-residing *Thra* KO cells (Figure 3A). The expression of osteoblast lineage marker *Bglap* was upregulated 2.09-fold by T4 in WT mice only (Figure 3B), while mRNA levels of common osteocyte markers *Dmp1* and *Sost* did not differ between the groups (Figure 3C and D). Interestingly, expression of genes encoding for major osteoclast enzymes TRAP (*Acp5*, 2.24-fold) and cathepsin K (*Ctsk*, 2.34-fold) was elevated in hyperthyroid WT bone tissue, but not *Thra*-derived bones (Figure 3E and F). We also checked the expression of early osteoclast marker nuclear factor of activated T-cells cytoplasmic 1 (*Nfatc1*) as well as *Rankl* and osteoprotegerin (*Opg*), however, did not detect significant changes between the groups (Figure 3G-J). Conclusively, these data suggest *Thra* might be a prerequisite for the expression of bone resorption-associated genes in osteoclasts, osteocytes and/or other bone resident cells, when treated with TH.

### *Thra* loss forestalls osteoclast marker expression in T_3_-treated late osteoblasts/osteocytes

To explore the role of *Thra* in primary osteocytes, we used WT and *Thra*^0/0^ mice derived late osteoblasts/osteocytes to study their TH responsiveness in an isolated condition. Expression of osteocyte marker genes, in particular, dentin matrix acidic phosphoprotein 1 (*Dmp1*, average CT = 24.0), phosphate regulating endopeptidase X-linked (*Phex*, average CT = 30.4), and matrix extracellular phosphoglycoprotein (*Mepe*, average CT = 27.3), confirmed an osteocytic character of these cells after 28 d of differentiation.

**Figure 4:**
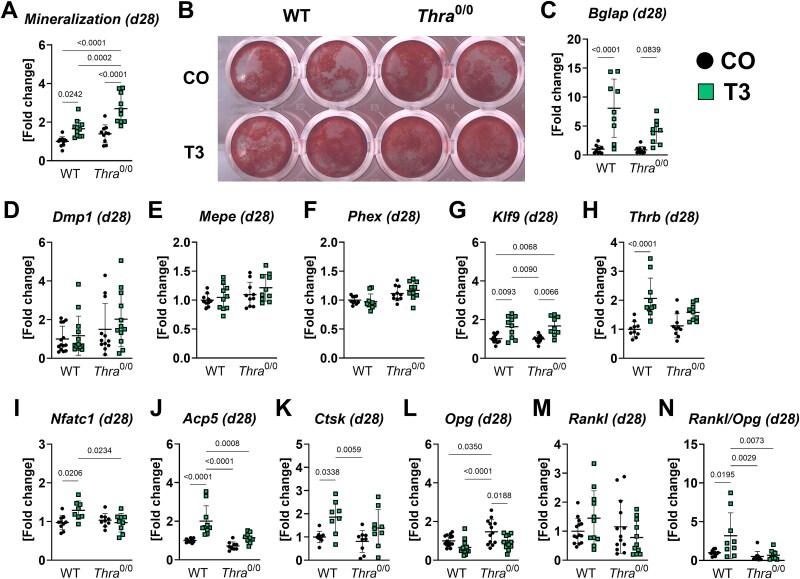
*Thra*-deficient late osteoblasts/osteocytes do not upregulate osteoclast marker genes with T_3_. Mesenchymal stromal cells from *Thra* KO mice (*Thra*^0/0^) and respective WT littermates were used to grow late osteoblasts/osteocytes over 28 d under cell culture conditions. To evaluate the mineralization capacity, cells were differentiated and simultaneously treated with 100 nM T_3_ (T3) over 28 d. (A) Mineralization capacity was evaluated by Alizarin Red staining and (B) a representative picture of Alizarin Red-stained cells is shown. Further, we treated late osteoblasts/osteocytes over 48 h with 100 nM T_3_ and used real-time quantitative PCR to assess the expression of (C) osteoblast lineage marker osteocalcin/bone gamma-carboxyglutamate protein (*Bglap*), (D) osteocyte marker dentin matrix acidic phosphoprotein 1 (*Dmp1*), (E) phosphate regulating endopeptidase X-linked (*Phex*), (F) matrix extracellular phosphoglycoprotein (*Mepe*), (G) TH target gene krueppel like factor 9 (*Klf9*), and (H) thyroid hormone receptor β (*Thrb*). To check for osteoclastic features, expression of osteoclast marker genes (I) nuclear factor of activated T-cells cytoplasmic 1 (*Nfatc1*), (J) TRAP-encoding gene acid phosphatase 5, tartrate resistant (*Acp5*), and (K) cathepsin K (*Ctsk*) was tested. In addition, mRNA levels of (L) the receptor activator of NF-κB ligand (*Rankl*) and (M) osteoprotegerin (*Opg*) were quantified and the (N) *Rankl/Opg* ratio was calculated. Each dot indicates an individual mouse. Mineralization: *N* = 11 per group. Real-time qPCR: WT/CO: *N* = 10; WT/T3: *N* = 9; *Thra*^0/0^/CO: *N* = 10; *Thra*^0/0^/T3: *N* = 10. The horizontal lines represent the mean ± SD. Statistical analysis was performed by 2-way ANOVA and *p*-values are shown within the graph.

Mineralization capacity of WT and *Thra*-deficient late osteoblasts/osteocytes was increased with T_3_ 1.66-fold vs WT/CO and 1.94-fold vs *Thra*^0/0^/CO, respectively (Figure 4A and B). The mineralization was even more pronounced in T_3_-treated KO cells (*Thra*^0/0^/T_3_ 1.61-fold vs WT/T_3_) (Figure 4A). Further, we found an upregulated *Bglap* expression in both hyperthyroid WT (8.09-fold) and *Thra* KO (4.64-fold, *p* = .08) cells as compared with respective untreated controls (Figure 4C), while osteocyte marker genes *Dmp1*, *Mepe*, and *Phex* were not altered comparing the 4 groups (Figure 4D-F). T_3_-induced *Klf9* expression in both groups verifying a successful treatment (Figure 4G). Interestingly, we found an upregulation of the other TR gene, *Thrb*, exclusively in T_3_-treated WT late osteoblasts/osteocytes (2.07-fold vs WT/T_3_, Figure 4H). Furthermore, mRNA levels of osteoclast marker genes *Nfact1*, *Acp5*, and *Ctsk* (1.29-/2.01-/1.86-fold vs WT/T_3_) were only elevated with T_3_ in WT, but not *Thra*-deficient cells (Figure 4I-K). With regards to osteoblast/osteoclast coupling, we observed a higher expression of *Opg*, a major osteoclast inhibitor, in untreated *Thra* KO vs WT cells (*Thra*^0/0^/CO 1.47-fold vs WT/CO) that declined with T_3_ treatment (*Thra*^0/0^/CO −36.16% vs *Thra*^0/0^/T_3_, Figure 4L). While *Rankl* mRNA levels were unchanged comparing all groups, the overall *Rankl/Opg* ratio significantly increased in T_3_-treated WT cells only (3.20-fold vs WT/T_3_, Figure 4M and N). Taken together, these in vitro data reveal that *Thra*-deficient late osteoblasts/osteocytes are T_3_ responsive; however, the upregulation of osteoclast genes, *Thrb*, and the *Rankl/Opg* ratio seems to be *Thra*-dependent.

## Discussion

Thyroid hormones are important regulators of bone health in adults, and thus thyroid diseases, in particular hyperthyroidism, can have detrimental effects on bone mass, microarchitecture, and strength. Thyroid hormone actions are mediated by specific TRs with TRα1 being the most prominent one in bone tissue. Still, the role of TRα in hyperthyroidism-driven bone loss and fragility has been not fully uncovered. Here, we used *Thra*^0/0^ mice and derived primary osteocytes lacking all TRα isoforms to assess their bone/cell phenotype after TH treatment.

Hyperthyroid WT mice displayed both trabecular and cortical bone loss at the spine and femur, while hyperthyroid *Thra*^0/0^ mice showed primarily reduced trabecular bone volume in load-bearing femurs. In line, Monfoulet et al. showed rapid trabecular bone loss at the femur of *Thra*^0/0^ mice when treated with T4 over 3 wk, while they did not comment on the cortical bone.^46^ Here, we found preserved cortical bone volume, mineral density, and thickness in *Thra*-deficient mice despite T4 treatment implying that TH actions might be either site- and/or cell-specifically dependent on *Thra* expression. It is known that TR and their target genes can have a tissue- and developmental stage-dependent expression.^47^^,^^48^ Given that TR antibodies lack sufficient sensitivity, TR protein expression in osteoclasts and osteocytes has not been reported yet.^3^ Moreover, in our previous study, we found that T_3_ treatment alone did not influence osteoclast number or activity in vitro, whereas conditioned media from T_3_-treated WT osteoblasts stimulated late osteoclast marker expression.^18^ Thus, we can only speculate whether functional TR in either osteoclasts and/or osteocytes might directly mediate TH actions in vivo or whether observed effects on trabecular vs cortical bone mass are driven by surrounding cells of the osteoblast lineage. In line with the preserved cortical BMD and thickness, bone strength tested at the femoral midshaft was unaffected by hyperthyroidism in *Thra*-deficient mice identifying *Thra* as a preserver of bone strength under TH excess.

Moreover, TRAP serum levels and numbers of TRAP-expressing osteoclasts were not significantly increased in hyperthyroid *Thra*^0/0^, but WT mice, indicating that bone resorption was less affected in T4-treated KO than WT animals. Tartrate-resistant acid phosphatase is a common bone resorption marker and reflects the activity of osteoclasts in patient sera.^49^ In the past, TRAP activity has been also reported in osteocytes^50^ and was now attributed to the process of osteocytic osteolysis.^36^^,^^37^^,^^51^^,^^52^ In our previous study, we found a higher TRAP activity in osteocytes and 1.14-fold larger osteocyte lacunae, demonstrating a mild form of osteocytic osteolysis in C57BL/6 mice with T4 treatment.^10^ Here, we detected a robust TRAP activity in trabecular and cortical osteocytes in load-bearing femurs of hyperthyroid WT mice, but not *Thra* KO mice. Accordingly, expression of *Trap* and *Ctsk*, encoding for cathepsin K, another potential contributor to osteocytic osteolysis,^53^ was upregulated only in WT bone tissue upon T4 treatment. These data suggest a *Thra*-dependent TRAP activation and potentially bone resorptive activity of osteocytes under TH excess conditions. Furthermore, studies show that impaired perilacunar canalicular remodeling can contribute to overall bone loss^10^^,^^39^^,^^40^ and increase bone fragility even when bone mass is normal.^54^ Thus, fewer TRAP^+^ osteocytes might be associated with stronger bones in T4-treated *Thra*-deficient compared to WT animals.

In vitro, we found that *Thra*-deficient late osteoblasts/osteocytes are still TH responsive as shown by TH target gene *Klf9* upregulation, consistent with the findings from our previous study on *Thra*^0/0^ early and mature osteoblasts.^18^ In contrast, we did not observe enhanced differentiation and activity comparing untreated WT vs *Thra* KO osteocytes implying that *Thra* might have a developmental stage-dependent function through osteoblast to osteocyte transition. In accordance to the gene expression patterns in bone tissue, *Trap*, *Ctsk*, and additionally *Nfatc1*, newly identified as a regulator of osteocytic osteolysis,^55^ were upregulated in vitro by T_3_ in osteocytes in a *Thra*-dependent manner. In line with our previous findings in mature osteoblasts,^18^  *Thra* deletion inhibited the T_3_-driven increase in *Rankl/Opg* ratio in osteocytes that would promote bone resorption by osteoclasts. While these effects reached statistical significance in vitro, only a non-significant trend towards increased *Rankl* expression and an elevated *Rankl/Opg* ratio was observed in bone tissue from hyperthyroid WT mice. Overall, these data indicate a potential role for *Thra* in regulating bone resorption-associated genes in TH-stimulated osteocytes.

Interestingly, *Thrb* expression was not upregulated with T_3_ treatment in *Thra*-depleted cells. Due to the intricate nature of TH signaling within the nucleus, encompassing transcriptional activators and repressors, chromatin remodeling, and co-receptors, the precise molecular mechanisms remain largely uncovered. Unbound TR can function as transcriptional repressors, and an imbalanced intracellular TRα:TRβ ratio can alter the transcriptional dynamics of TH target genes.^3^^,^^56–58^ Therefore, it remains unclear whether the observed changes in osteocytes are primarily driven by the complete absence of all *Thra* isoforms or by the relative predominance of *Thrb* isoforms.

In addition to the novel information this study provides, it has potential limitations. While pituitary production of growth hormone and circulating estrogen levels, two major drivers of bone remodeling besides THs, were not affected in *Thra*^0/0^ mice,^59^^,^^60^ effects on other primarily TRα-expressing tissues,^61^ and their production of osteomodulatory cytokines and hormones, such as glucocorticoids, irisin, insulin-like growth factor 1, and adiponectin have not been investigated in this study. Further, given the global *Thra* KO, we cannot attribute the observed effects to a single skeletal cell type. Thus, bone cell-specific *Thra*- and *Thrb*-floxed KO mouse models would represent valuable tools for elucidating the specific roles of the individual TR in skeletal cells and the regulation of bone remodeling in vivo. In addition, alternative *Thra* mutants could be used to elucidate the role of TRα1 vs TRα2 (TRα1^−/−^; TRα2^−/−^) in or without the presence of the nonfunctional isoforms TRΔα1 and TRΔα2 (*Thra*^−/−^); however, these transgenic mice present an altered TH status that could interfere with T4 treatment. Nevertheless, this study is the first to unravel the bone compartment-specific role of *Thra* in hyperthyroid male mice. Another limitation of the current study is the exclusive use of male mice. Future studies will be required to determine whether the observed effects are modulated by female sex hormones.

In conclusion, global *Thra* KO preserves cortical bone integrity in hyperthyroid male mice by mitigating bone resorption. Additional studies are required to clarify how individual Thra isoforms in specific bone cell types contribute to hyperthyroidism-related bone loss.

## Supplementary Material

Supplemental_Methods_Brinkmann_et_al_ziag033

## Data Availability

All data are available from the corresponding author upon reasonable request.
