# Supplementary material for: Thra knockout protects male mice from hyperthyroidism-driven cortical bone loss by mitigating bone resorption
Source: JBMR Plus. 2026 Mar 6;10(5):ziag033. doi: 10.1093/jbmrpl/ziag033 (PMC13050503; doi:10.1093/jbmrpl/ziag033)
Supplement: Supplemental_Methods_Brinkmann_et_al_ziag033 [file supplemental_methods_brinkmann_et_al_ziag033.pdf]

SUPPLEMENTAL TABLE

Supplemental table S1. Primer sequences for mice used for quantitative real-time PCR.

Gene	RefSeq #	Sense	Antisense
Acp5	NM_001102405	ACTTGCGACCATTGTTAGCC	AGAGGGATCCATGAAGTTGC
beta-Actin	NM_007393	GATCTGGCACCACACCTTCT	GGGGTGTTGAAGGTCTCAAA
Bglab	NM_001032298	GCGCTCTGTCTCTCTGACCT	ACCTTATTGCCCTCCTGCTT
Ctsk	NM_007802	AAGTGGTTCAGAAGATGACGGGAC	TCTTCAGAGTCAATGCCTCCGTTC
Dio3	NM_172119	ATTTTGAGCGCCTCTACGTC	ATCATAGCGCTCCAACCAAG
Dmp1	NM_016779	AGTGAGTCATCAGAAGAAAGTCAAGC	CTATACTGGCCTCTGTCGTAGCC
Klf9	NM_010638	GGCTGTGGGAAAGTCTATGG	AAGGGCCGTTCACCTGTATG
Mepe	NM_053172	GCAGCACCAATTTCAGGGAA	TTTCTCTCGAGGCCACTTGT
Nfatc1	NM_016791	GTTCCTTCAGCCAATCATCC	GGAGGTGATCTCGATTCTCG
Opg (Tnfrsf11b)	NM_008764	CCTTGCCCTGACCACTCTTA	ACACTGGGCTGCAATACACA
Phex	NM_011077	GGAAGAAAACCATTGCCAATTATT	CGCCTGCTGAGGTTTGGA
Rankl (Tnfsf11)	NM_011613	CCAAGATCTCTAACATGACG	CACCATCAGCTGAAGATAGT
Sost	NM_024449	CGGAGAATGGAGGCAGAC	GTCAGGAAGCGGGTGTAGTG
Thrb	NM_001113417	CAGGACTGGAAGCTGGTAGG	AGTGGCCTGGATAAGGTGTG
